# Sex steroid and cognitive function among community-dwelling older men with or without vascular risk factors: a cross-sectional study

**DOI:** 10.1186/s12877-024-04727-6

**Published:** 2024-02-13

**Authors:** Shuning Tang, Limei Huang, Fangting Lin, Xiuqin Chen, Yunhui Wang, Jixiang Xu, Yujie Wang, Junling Gao, Qianyi Xiao

**Affiliations:** 1 Department of Preventive Medicine and Health Education, School of Public Health, The Key Laboratory of Public Health Safety of Ministry of Education, Fudan University, 200032 Shanghai, China; 2 Health Communication Institute, Fudan University, 200032 Shanghai, China; 3 Songjiang Center of Disease Prevention and Control, 201620 Shanghai, China; 4 Songjiang District Xinqiao Town Community Health Service Center, 201600 Shanghai, China

**Keywords:** Sex steroid, Cognitive function, Vascular risk factors, Older men

## Abstract

**Background:**

The relationship of testosterone and estradiol concentrations with cognitive function among community-dwelling older men was inconclusive. To examine the association of serum testosterone and estradiol concentrations with cognitive function in older men with or without vascular risk factors (VRFs).

**Methods:**

This cross-sectional study consisted of 224 community-dwelling men aged 65–90 years in the Songjiang District of Shanghai, China. Serum testosterone and estradiol were measured by electrochemiluminescence immunoassay. The following five factors were defined as VRFs in this study: obesity, history of hypertension, diabetes, stroke, and coronary heart disease. Multivariable linear regression was used to examine the association of testosterone and estradiol with the Mini-Mental State Examination (MMSE) in participants with or without VRF. Restricted cubic spline (RCS) regression was performed to account for the nonlinearity of these associations.

**Results:**

An inverted “U” shaped non-linear relationship was found between testosterone concentration and MMSE score in men with one VRF (*P* overall =.003, non-linear *P* =.002). Estradiol showed an inverted “U” shaped non-linear relationship with MMSE score independent of VRFs (men without VRF, *P* overall =.049, non-linear *P* =.015; men with one VRF, overall *P* =.007, non-linear *P* =.003; men with two or more VRFs, overall *P* =.009, non-linear *P* =.005).

**Conclusion:**

In older men, an optimal level of sex steroid concentration may be beneficial to cognitive function and the VRFs should be considered when interpreting the relationship between sex steroid and cognitive function.

## Background

Cognitive deficit accumulates with age and poses an urgent healthcare issue among seniors. It is estimated that approximately 6.97% of older adults aged 50 and over in the community have dementia [1]. Evidence has suggested that the risk factors and progression of dementia may be gender-dependent [2]. Epidemiological studies showed that women had a higher prevalence of Alzheimer’s disease (AD) than men, whereas men had a higher prevalence of vascular dementia (VD) [1]. Biological studies also indicated that sexual differentiation of the brain during development could lead to various permanent structural and functional differences between adult male and female brains [3] due to sex steroids’ organizational effects [4].

The loss of the effect of estrogen and androgen caused by normal aging could contribute to a series of aging changes that influence the pathogenesis of dementia [5, 6]. However, in men, the association between sex steroids and cognitive function in previous population-based studies has not reached a consensus. Most studies supported the beneficial role of testosterone in cognitive function, including the associations of lower free testosterone with an increased AD risk [7, 8] and worse working memory in older men [9]. Randomized intervention trials also demonstrated that testosterone supplementation can moderately improve men’s overall cognitive function [10] and specific cognitive domains, such as spatial memory [11, 12], visuospatial function [13], verbal memory [12, 14] and structural ability [12]. However, higher levels of serum total and free testosterone were found in AD patients than in controls [15] and high levels of testosterone hindered the language fluency practice in healthy German older men [16]. Concerning estradiol, epidemiologic studies showed that higher estradiol levels (total and bioavailable) were associated with an increased risk of cognitive decline [17], but a better spatial memory [18], visual memory [19], spatial span performance and verbal memory [18]. Recently, Ross J. Marriott et al. explored the nonlinear relationship between testosterone and dementia and found that low testosterone is associated with a greater risk of dementia [8], but this study lacks estradiol data.

The vascular risk factors (VRFs) could directly increase the susceptibility to cognitive decline and dementia [20], or the effect can be mediated via cardio and cerebrovascular disease [21]. Besides, lower endogenous testosterone was found to be associated with an increased risk of cardiovascular disease. However, few studies took VRFs into account in analyzing the association between sex steroids and cognition. We hypothesized that sex steroids may have diverse effects on cognitive function in different groups of individuals. The purpose of this preliminary study is therefore to examine our hypothesis among community-dwelling older men.

## Methods

### Sampling and procedures

This cross-sectional study was performed in Xinqiao Street, Songjiang District, Shanghai, China, from July 2021 to September 2021. Communities with an elderly (≥ 60) population greater than 200 on Xinqiao Street were chosen. Next, one community was randomly selected among these selected communities and the list of home addresses was extracted. Informed consent forms were distributed to the older people on the list through the neighborhood committee, inviting them to participate in the investigation. All men recruited in the study were registered residents of Songjiang Community in Shanghai, aged ≥ 60 years old, able to communicate and undergo physical and cognitive examinations and consent to provide blood samples and sign the informed consent form. This study excluded men with severe schizophrenia or mental retardation, prostate cancer, orchiectomy. Participants were interviewed via a questionnaire survey for their demographic characteristics, including birth date, education level, depression, and lifestyles (smoking, drinking, and physical activity). The blood sample collection was completed in Songjiang District Xinqiao Town Community Health Service Center.

### Measures

#### Cognition assessment

Global cognitive ability was assessed using the Mini-Mental State Examination (MMSE). The test is composed of 30 items to assess orientation, immediate and delayed recall, attention, language, and visuospatial ability. MMSE scores range from 0 to 30 and greater indicate better cognitive function. The MMSE testing was performed through face-to-face interviews with interviewers in a quiet environment. All interviewers were systematically trained by a neuropsychologist before the study.

#### Sex steroids

Fasting blood sample of each participant was collected and the serum was kept at -80℃ until analyzed. The electrochemiluminescence immunoassay (Cobas e411, Roche Diagnostics GmbH, Mannheim, Germany) was used to measure serum levels of estradiol and testosterone. The measuring range of estradiol was 5-3000pg/mL; the intra-assay coefficient of variation (CV) was 3.0% at 93.30 ± 2.82 pg/mL and 5.9% at 456.34 ± 26.89 pg/mL; the interassay CV was 3.3% at 93.30 ± 2.82 pg/mL and 5.1% at 456.34 ± 26.89 pg/mL. The measuring range of testosterone was 0.025 − 15.000 ng/mL; the intra-assay CV was 3.6% at 2.44 ± 0.09 ng/mL and 1.8% at 6.01 ± 0.11 ng/mL; the interassay CV was 3.4% at 2.44 ± 0.09 ng/mL and 1.9% at 6.01 ± 0.11 ng/mL.

#### Covariates and stratification factor

Data on birthday date, and education levels (below primary school, primary school, junior high school, and senior high school, and above) were collected from participants’ self-reports. Height, weight, and waist circumference (WC) were measured. Height and weight were used to calculate body mass index (BMI). Obesity was defined as a BMI ≥ 28.0 according to the criteria of weight for adults published by the National Health Commission of China. Medical histories (hypertension, diabetes, stroke, coronary heart diseases [CHDs], and depression diagnosed by physicians) were reported by participants and then verified by their medical records. Smoking status was determined through two inquiries: “Have you smoked 100 cigarettes in your lifetime?” and “Have you smoked within the past 30 days?” Individuals who had both smoked 100 cigarettes in their lifetime and had smoked within the last 30 days were classified as smokers. Drinking status was divided into two categories: those who drank and those who had never consumed alcohol. To evaluate physical activity (PA), two questions were posed: “In the last week, how many days did you engage in moderate-intensity physical activities, such as playing badminton, brisk walking, playing table tennis, and square dancing? (None, 1–2 times, 3–4 times, 5–6 times, 7 times or more)” and “How long did you spend on each occasion of physical activity? (50 minutes) [22]”, and the product of these two responses was used to calculate the weekly duration of PA. By recommendations from the American Heart Association [23] and current guidelines for physical activity [24], PA was categorized as inactive (PA < 150 min/ week) or active (PA ≥ 150 min/ week). Depression was assessed using the Patient Health Questionnaire-9 (PHQ-9), which comprises nine questions. Scores for each question ranged from 0 (not at all) to 1 (several days), 2 (more than half of the days), and 3 (nearly every day). Depression was defined as a PHQ-9 score equal to or greater than 5 [25]. The following five factors were defined as VRFs in this study: obesity, history of hypertension, diabetes, stroke, and CHDs [21].

### Statistical analysis

Demographic characteristics of the participants were shown using descriptive statistical methods. Continuous variables are presented as mean (SD, Standard Deviation) and categorical variables are presented as number of cases (n) and frequency (%). Comparisons were performed in groups with different VRFs carrier statuses (without VRF, with one VRF, and with two or more VRFs), using the ANOVA test for normally distributed continuous data, the Kruskal-Wallis test for non-normally distributed continuous data, and χ^2^ tests for dichotomous variables. The association between testosterone and estradiol was tested by a linear regression adjusting for age and BMI. Multivariable linear regression models were used for analyzing the association between the exposure variables (testosterone and estradiol) and MMSE score in men without VRF, with one VRF, and with two or more VRFs, respectively. The residuals of models are verified by the Q-Q plots and conform to normal distributions. For the subgroup without VRF, the adjusted covariates in Model 1 included age, education, BMI, WC, and depression; in Model 2, additional testosterone (for estradiol) or estradiol (for testosterone) was added. For the subgroup with one VRF and two or more VRFs, the adjusted covariates in Model 1 included age, education, BMI, WC, depression, diabetes mellitus, hypertension, CHD, and stroke; in Model 2, additional testosterone (for estradiol) or estradiol (for testosterone) was added. The effects of the exposure variables were expressed in terms of standardized regression coefficients (β) and 95% Confidence Intervals (CI). Restricted cubic splines (RCS) were used to examine the non-linear association between exposure variables (testosterone and estradiol) and MMSE score stratified by VRFs. The adjustments in RCS analyses were the same as those in Model 2 in each group. RCS can reflect the overall association and non-linear results of generalized linear regression in images. The reference value (y = 0) was set at the 10th percentile. *P*-overall values are for the overall trend and *P*-non-linear values are for the nonlinear correlation, with a threshold of *P* <.05 regarded as significant. All analyses were conducted in R version 4.2.0 and SPSS version 25.0.

## Results

### Participants’ characteristics

**Fig. 1:**
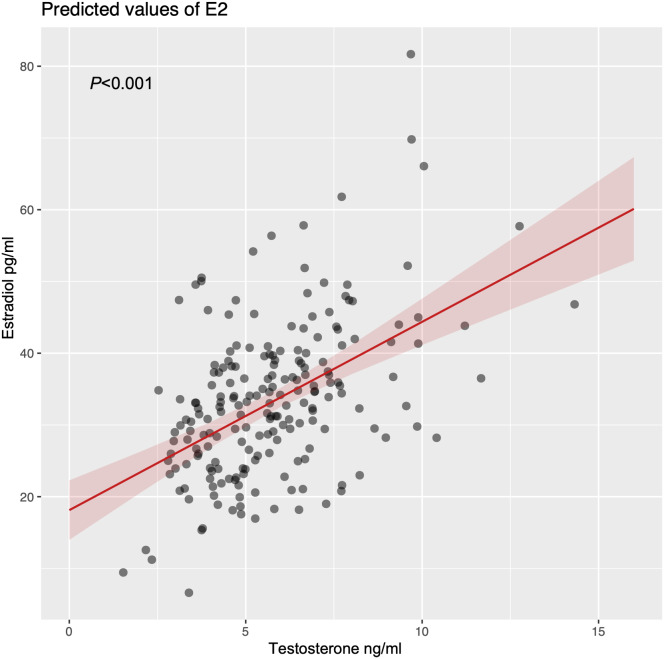
Association between testosterone and estradiol by linear regression. The testosterone and estradiol test data were obtained from 224 men. The lines represent the estradiol concentration (solid lines) and 95% confidence intervals (shading)

A total of 289 men were included in this study, among which 65 were excluded due to a lack of blood samples (*n* = 61) and sex steroids below the lowest limit of detection (*n* = 4). Finally, 224 participants were included in the analysis dataset, which included 77 men without VRF, 103 men with one VRF, and 44 men with two or more VRFs. As shown in Table 1, the mean age of the participants was 71.8 (SD 5.1) years and the mean MMSE score was 26.6 (SD 4.5). Half of the participants had primary school qualifications. Compared to men without VRF, men with one and two or more VRFs had higher BMI, WC, and lower testosterone levels, and were more likely to have depression (all, *P* <.05). There was no significant difference in age, MMSE score, smoking, drinking, physical activity and estradiol among groups with different VRFs carrier status (without VRF, with one VRF, and with two or more VRFs) (all, *P* >.05). In this study, testosterone concentration was found to be positively correlated to estradiol (β = 3.09, Std. error = 0.33, *P* <.001, Fig. 1) after adjusting age and BMI.

**Table 1:** The characteristics of study participants

Characteristics ^a, b^	All	Men without VRF	Men with one VRF	Men with two or more VRFs	P- value
	*N* = 224	N (%) = 77 (34.4)	N (%) = 103 (46.0)	N (%) = 44 (19.6)	
**Sociodemographic**					
Age, mean (SD)	71.8 (5.1)	71.3 (4.5)	72.0 (5.3)	72.4 (5.9)	0.50
Education, n (%)					
Below primary school	36 (16.1)	11 (14.3)	20 (19.4)	5 (11.4)	0.08
Primary school	106 (47.3)	34 (44.2)	48 (46.6)	24 (54.5)	
Junior high school	62 (27.7)	19 (24.7)	29 (28.2)	14 (31.8)	
Senior high school and above	20 (8.9)	13 (16.9)	6 (5.8)	1 (2.3)	
BMI, mean (SD)	24.5 (3.2)	23.01 (2.4)	24.7 (2.7)	26.5 (4.3)	< 0.001
WC, mean (SD)	85.4 (8.8)	82.0 (7.5)	85.7 (8.0)	90.5 (9.8)	< 0.001
MMSE, mean (SD)	26.6 (4.5)	26.5 (5.2)	26.6 (4.4)	26.6 (3.8)	0.99
Depression, yes, n (%)	18 (8.0)	3 (3.9)	6 (5.8)	9 (20.5)	0.003
**Lifestyle**					
Smoking, yes, n (%)	84 (37.5)	33 (42.9)	40 (38.8)	11 (25.0)	0.14
Drinking, yes, n (%)	38 (17.0)	12 (15.6)	18 (17.5)	8 (18.2)	0.92
Physical activity, active, n (%)	112 (50.0)	36 (46.8)	52 (50.5)	24 (54.5)	0.71
**VRFs**					
Obesity, yes, n (%)	27 (13.1)	-	7 (7.6)	20 (48.8)	< 0.001
Hypertension, yes, n (%)	123 (54.9)	-	82 (79.6)	41 (93.2)	< 0.001
Diabetes, yes, n (%)	23 (10.3)	-	6 (5.8)	17 (38.6)	< 0.001
CHDs, yes, n (%)	18 (8.0)	-	7 (6.8)	11 (25.0)	< 0.001
Stroke, yes, n (%)	11 (4.9)	-	1 (1.0)	10 (22.7)	< 0.001
**Hormone Variables**					
Testosterone (ng/mL), mean (SD)	5.6 (2.1)	6.1 (2.1)	5.5 (2.3)	5.1 (1.8)	0.039
Estradiol (pg/mL), mean (SD)	32.5 (11.6)	33.0 (10.9)	33.7 (11.7)	30.9 (9.9)	0.36

a. Continuous variables (age, BMI, WC, MMSE, estradiol, and testosterone) represented as mean (SD, Standard Deviation); other variables as numbers (percentages) per category

b. VRFs, vascular risk factors; BMI, body mass index (kg/m^2^); WC, waist circumference (cm); MMSE, The Mini-Mental State Examination; CHDs, coronary heart diseases

### Association between testosterone and MMSE score stratified by VRFs

**Fig. 2:**
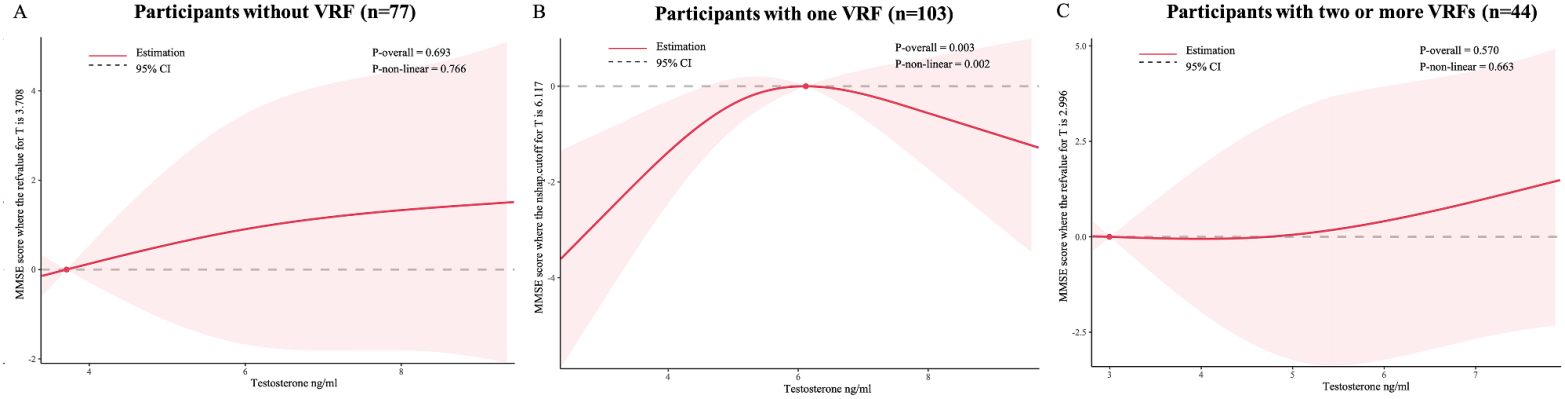
RCS showed the association between testosterone and MMSE score. Among participants without VRF (**A**), RCS was adjusted for age, education, BMI, WC, depression, and estradiol. Among participants with one VRF (**B**) and two or more VRFs (**C**), RCS was adjusted for age, education, BMI, WC, hypertension, diabetes, CHDs, stroke, depression, and estradiol. The lines represent the MMSE score (solid lines) and 95% confidence intervals (shading). The reference values (y = 0, The y-axis is compressed) were set at the 10th percentile, and the knots were set at the 10th, 50th, and 90th percentiles of the level of testosterone

As shown in Table 2, among participants with one VRF, testosterone in the third quartile was associated with a higher MMSE score compared with testosterone in the lowest quartile (Q1) both in Model 1 (Q3 vs. Q1, β = 3.02, 95% CI = 0.83–5.21, *P*-trend =.027) and Model 2 (Q3 vs. Q1, β = 2.74, 95% CI = 0.44–5.03). However, no significant association between testosterone and cognition was observed in participants without VRF or in participants with two or more VRFs. RCS analysis showed that the inverted “U” shaped non-linear relationship between testosterone and MMSE score was only significant in those with one VRF (overall *P* =.003, non-linear *P* =.002, Fig. 2B).

**Table 2:** The relationship between testosterone/estradiol levels and MMSE score in subgroups stratified by VRFs

Subgroups	Q1 ^a^	Q2 ^a^	Q3 ^a^	Q4 ^a^	P- trend
	(The lowest)	β (95% CI)	β (95% CI)	β (95% CI)	
***Participants without VRF (N = 77)*** ^b^					
Testosterone					
Model 1	Ref.	-1.28 (-5.10, 2.54)	0.98 (-2.34, 4.30)	0.18 (-3.03, 3.39)	0.65
Model 2	Ref.	-1.32 (-5.25, 2.61)	0.72 (-3.01, 4.45)	-0.19 (-0.91, 3.52)	0.83
Estradiol					
Model 1	Ref.	1.91 (-1.39, 5.20)	0.95 (-2.44, 4.33)	1.26 (-1.83, 4.34)	0.56
Model 2	Ref.	1.65 (-1.84, 5.14)	0.49 (-3.43, 4.40)	1.06 (-2.47, 4.59)	0.68
***Participants with one VRF (N = 103)*** ^c^					
Testosterone					
Model 1	Ref.	1.96 (-0.07, 4.00)	3.02 (0.83, 5.21)**	2.34 (-0.07, 4.75)	0.027
Model 2	Ref.	1.57 (-0.50, 3.65)	2.74 (0.44, 5.03)*	2.21 (-0.52, 4.95)	0.07
Estradiol					
Model 1	Ref.	1.09 (-1.29, 3.48)	2.52 (0.40, 4.65)*	1.15 (-1.09, 3.38)	0.17
Model 2	Ref.	0.34 (-2.16, 2.84)	1.75 (-0.46, 3.96)	0.16 (-2.39, 2.72)	0.66
***Participants with two or more VRFs (N = 44)*** ^c^					
Testosterone					
Model 1	Ref.	1.44 (-1.42, 4.30)	0.99 (-3.17, 5.15)	3.29 (-0.04, 6.62)	0.07
Model 2	Ref.	1.82 (-0.93, 4.58)	0.82 (-3.18, 4.81)	3.01 (-0.33, 6.34)	0.19
Estradiol					
Model 1	Ref.	2.71 (-0.23, 5.65)	4.17 (1.20, 7.14)*	2.46 (-1.20, 6.12)	0.052
Model 2	Ref.	3.03 (0.08, 5.99)	3.82 (0.82, 6.81)*	1.58 (-2.22, 5.38)	0.15

a. Quartile boundaries for testosterone Q1/2 4.1 ng/ml, Q2/3 5.6 ng/ml, and Q3/4 6.8ng/ml; quartile boundaries for estradiol Q1/2 25.7 pg/ml, Q2/3 32.7 pg/ml, and Q3/4 38.9 pg/ml

b. In the subgroup without VRF, Model 1 adjusted for age, education, BMI, WC, and depression; Model 2 additionally adjusted for testosterone (for estradiol) or estradiol (for testosterone)

c. In the subgroup with one VRF and two or more VRFs, Model 1 adjusted for age, education, BMI, WC, depression, diabetes mellitus, hypertension, coronary heart disease, and stroke; Model 2 adjusted for testosterone (for estradiol) or estradiol (for testosterone)

*P <.05, ** *P* <.01

### Association between estradiol and MMSE score stratified by VRFs

**Fig. 3:**
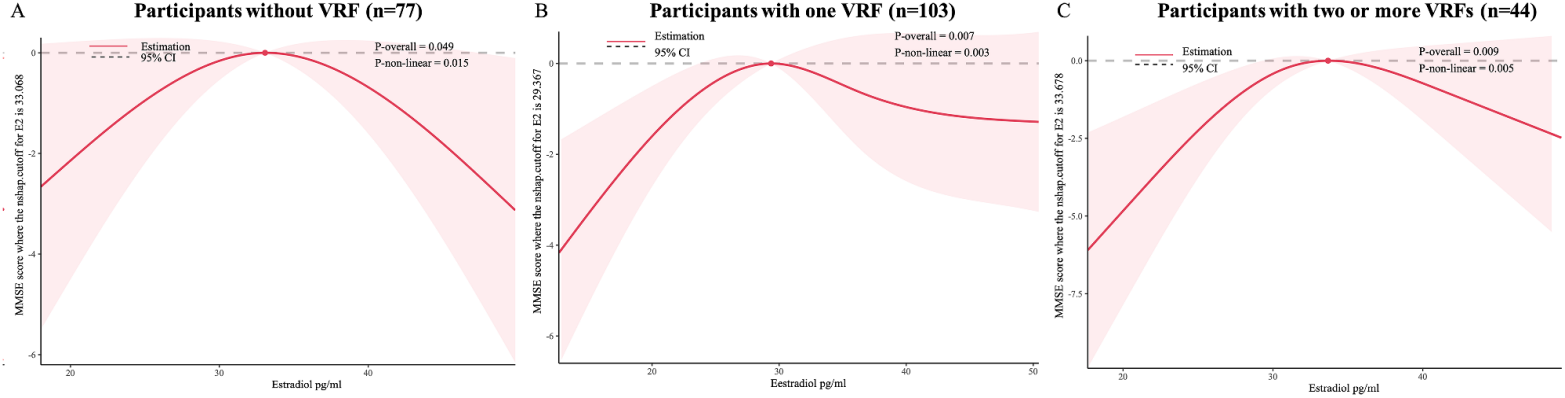
RCS showed the association between estradiol and MMSE score. Among participants without VRF (**A**), RCS was adjusted for age, education, BMI, WC, depression, and testosterone. Among participants with one VRF (**B**) and two or more VRFs (**C**), RCS was adjusted for age, education, BMI, WC, hypertension, diabetes, CHDs, stroke, depression, and testosterone. The lines represent the MMSE score (solid lines) and 95% confidence intervals (shading). The reference values (y = 0, The y-axis is compressed) were set at the 10th percentile, and the knots were set at the 10th, 50th, and 90th percentiles of the level of estradiol

Among men with one VFR, men with estradiol in the third quartile had a 2.52 point of MMSE improvement compared to men with estradiol in the lowest quartile (Q1) in Model 1 (Q3 vs. Q1, β = 2.52; 95% CI = 0.40–4.56; Table 2). This association was not significant when additionally adjusted for testosterone in Model 2. Among participants with two or more VRFs, estradiol in the third quartile was associated with higher MMSE score compared with estradiol in the lowest quartile (Q1) both in Model 1 (Q3 vs. Q1, β = 4.17, 95% CI = 1.20–7.14; Table 2) and in Model 2 (Q3 vs. Q1, β = 3.82; 95% CI = 0.82-6.81; Table 2). In RCS analyses, estradiol showed an inverted “U” shaped non-linear relationship with MMSE score independent of VRFs (men without VRF, overall *P* =.049, non-linear *P* =.015, Fig. 3A; men with one VRF, overall *P* =.007, non-linear *P* =.003, Fig. 3B; men with two or more VRFs, overall *P* =.009, non-linear *P* =.005, Fig. 3C).

## Discussion

The present cross-sectional study demonstrated an inverted “U” shaped nonlinear association between testosterone concentration and MMSE score only among men with VRFs, and an inverted “U” shaped nonlinear relationship between estradiol and MMSE score in men independent of the VRFs.

In nonlinear analysis, the testosterone and MMSE score showed an inverted U-shaped pattern. The effect of an optimal level of testosterone in cognition was supported by previous epidemic studies. A study of 547 older men in the community showed that an optimal level of sex steroids may exist for some cognitive functions, including a U-shaped association of total testosterone with the Blessed Information-Memory-Concentration Test, and bioavailable testosterone with the “World” test [26]. A cross-sectional study in the UK also found that in healthy older men and women, optimal levels of testosterone were associated with better MMSE score at baseline [27]. Another study suggested that testosterone could reduce neuronal apoptosis, and this effect may require optimal values requires an optimal level, as supraphysiological testosterone levels increase the risk for apoptosis [28]. In addition, curvilinear associations were observed between testosterone and memory performance and processing capacity/speed, suggesting optimal sex hormone levels [29]. Several functional studies also indicated the protective role of testosterone in cognitive function. Testosterone was reported to stimulate microglia phagocytosis by removing the Aβ deposition and inhibiting the inflammatory response [30]. In the rat model of AD, testosterone was found to prevent cognitive decline through scavenging free radicals and enhancing synaptic plasticity [31, 32], as well as regulating neuronal bioenergetics by increasing mitochondrial function [33].

Testosterone has also been found to modulate vascular tone, increase erythropoiesis, and affect platelet aggregability and cardiomyocyte electrophysiology and contractile activity [34]. Testosterone administration improves endothelial-mediated vasodilation in some male animal models [35, 36]. Low androgen concentrations are associated with an increased risk of CHD in older men [37, 38], and there is a link between androgen deficiency and atherosclerosis [39, 40]. VRFs have also been recognized as risk factors for cognitive decline and dementia [41]. Therefore, we speculate that in elderly men with one VRF, the optimal range of testosterone may be beneficial for cognitive function partially through exhibiting protective effects on cardiovascular disease. However, in the context of multiple VRFs, testosterone is insufficient to present benefits for cognitive function. Although the benefit role of optimal endogenous testosterone in cognitive function in men with one VRF, the effectiveness of exogenous androgen replacement therapy is still unclear, and potential risks and side effects need to be considered [42]. The timing, dose, and route of administration are key factors determining the benefits and risks of hormone therapy [43].

Concerning estradiol, experimental studies showed a neuroprotective role of estradiol in the brain. For example, including the role of promoting neurite growth and establishing interneuronal communication [44–46]. Furthermore, both estradiol [47, 48] and testosterone [49] have been reported to regulate Tau phosphorylation, which is essential for its association with axonal microtubules and the regulation of axonal growth. Estradiol also regulates the interaction of Tau with neurotransmitter receptors [50]. However, in men, excessive estrogen can lead to the deposition of subcutaneous fat [51]. In addition, the European Association of Urology Guidelines 2020 reported that adipocytokines and estradiol in obesity, can suppress the hypothalamic-pituitary-gonadal axis [52], and inhibit testosterone production by reducing the release of pituitary gonadotropin. Combining our finding that estradiol showed an inverted “U” shaped non-linear relationship with MMSE score in community-dwelling older men, it is necessary to control obesity and maintain the optimal range of sex steroid levels in early prevention strategies for cognitive decline.

The effect of estradiol on cognitive function was controversial in previous epidemic studies. A 4-year follow-up study in the Netherlands (*N* = 242) found that an increase in serum estradiol might be associated with an increased risk of cognitive decline in older men [17]. However, The Einstein Aging Study found that high levels of total estradiol in older men are associated with better performance on a cue-based, controlled learning test of verbal memory [53]; and a 5-year follow-up study in Australia (*N* = 1705) did not showed any significant association between estradiol levels and cognitive decline in older men [54]. Our study may partially explain the conflicting results of previous studies, which suggest that there may be optimal levels of estradiol in cognitive function. The optimal level of estradiol in our study was 35.141 pg/mL for all participants. In line with our result, a study using mortality as an outcome measure found that men with abnormal low (< 12.90 pg/mL) and high (≥ 37.40 pg/mL) estradiol levels showed the highest death rate from congestive heart failure [55]. Research on supplementing with exogenous sex hormones demonstrated that the dosage was one of the key factors determining the benefits and risks of hormone therapy [56]. Here, our findings provide additional evidence for the nonlinear relationship between endogenous estradiol and cognitive function and possible optimal levels of estradiol.

Several limitations in this study should be acknowledged. First, this study is a cross-sectional study, which was unable to establish the directionality of the association between estradiol/testosterone levels and cognitive decline. Second, the subjects assessed in this study were from rural areas with low levels of education attainment, therefore, the results of this study should be carefully applied to urban populations when extrapolating. Third, we did not obtain the apolipoprotein E (*APOE)* genotype, which was previously reported that it may interact with sex steroids to impact cognitive function [57]. Fourth, we did not collect the hormone replacement therapy and gonadal surgery history of participants and further research should investigate this information and exclude relevant samples. Fifth, serum sex steroids can also be influenced by other potential factors, such as antidepressant dose, diet, etc. Finally, we did not collect the medical histories of prostate hyperplasia and did not test the dihydrotestosterone concentrations, which may be confounders of relationships between sex steroids and cognitive function in men. To date, the relationship between testosterone and prostatic hyperplasia is controversial. The androgen dependence of the first phases of prostate development has inspired the historical view that higher testosterone may be involved in benign prostatic hyperplasia (BPH) occurrence; however, recent evidence suggests a different scenario, that testosterone is not detrimental to the prostate [58]. With aging, the plasma level of testosterone decreases, as well as the testosterone/estrogen ratio, resulting in increased estrogen activity, which may facilitate the hyperplasia of the prostate cells. Another theory focuses on dihydrotestosterone (DHT) and the activity of the enzyme 5α-reductase, which converts testosterone to DHT [59]. A Danish nationwide cohort (1996–2016) found that men with BPH had persistently higher risk of AD and all-cause dementia compared with men in the general population [60]. Therefore, future studies are needed to confirm our findings with the information of prostatic hyperplasia and dihydrotestosterone.

## Conclusions

Results from this cross-sectional study provided preliminary evidence that among older men with VRFs, the optimal level of serum testosterone may be beneficial for cognitive function. The optimal level of estradiol concentration may benefit cognitive function both in men with and without VRF. Larger longitudinal studies are necessary to confirm the hypothesis.

## Data Availability

The data that support the findings of this study are available from the Department of Preventive Medicine and Health Education, School of Public Health, Fudan University, but restrictions apply to the availability of these data, which were used under license for the current study, and so are not publicly available. Data are however available from the authors Qianyi Xiao upon reasonable request and with permission of Preventive Medicine and Health Education, School of Public Health, Fudan University.

## Abbreviations

VRFs: Vascular Risk Factors
MMSE: the Mini-Mental State Examination
AD: Alzheimer’s disease
VD: vascular dementia
BMI: Body Mass Index
WC: waist circumference
PA: Physical Activity
CHDs: coronary heart diseases
CV: coefficient of variation
PHQ-9: the Patient Health Questionnaire-9
SD: Standard Deviation
RCS: Restricted Cubic Spline
CI: Confidence Intervals
SPSS: Statistical Product and Service Solutions
APOE: apolipoprotein E
